# Chemotyping of *Koelreuteria paniculata* Seed Cake with Bioactive and Feed Potential

**DOI:** 10.3390/plants14182873

**Published:** 2025-09-15

**Authors:** Veljko Šarac, Dragana Šunjka, Magdalena Pušić Devai, Tea Sedlar, Nedeljka Spasevski, Slađana Rakita, Danka Dragojlović, Zorica Tomičić, Katarina Šavikin, Jelena Živković, Ivana Čabarkapa, Mirjana Ljubojević

**Affiliations:** 1 Faculty of Agriculture, University of Novi Sad, 21000 Novi Sad, Serbia; 2 Institute of Food Technology, University of Novi Sad, Bulevar Cara Lazara 1, 21000 Novi Sad, Serbia; 3 Institute for Medicinal Plants Research “Dr Josif Pančić”, 11000 Belgrade, Serbia

**Keywords:** antimicrobial, amino acid, fatty acid, insecticidal, phenolic compounds

## Abstract

*Koelreuteria paniculata* is an amenity landscape tree whose seed extracts and cold-pressed oil are proven biopesticides and biodiesel feedstocks. However, the residual seed cake phytochemical profile has not been systematically assessed or evaluated for multifunctionality across pesticidal, fertilizing, and nutritional domains. Therefore, the aim of this study was to perform a comprehensive chemotyping of *K. paniculata* seed cake and evaluate its potential for use as a biopesticide, biofertilizer, and feed additive, contributing to sustainable and circular agricultural systems. Detailed analyses of the defatted seed cake included moisture, crude protein, crude ash, crude fat, and crude fiber determination, as well as amino acid and fatty acid composition determination, supplemented with HPLC and antioxidative capacity investigation. Results delivered a comprehensive chemotyping of *K. paniculata* seed cake, revealing a nutrient-rich profile with moderate protein (20.01%), substantial monounsaturated fatty acids (75.8%, mainly eicosenoic and oleic), and significant phenolic content, including ellagic acid, rutin, catechin, and gallic acid. Antioxidant assays (DPPH and ABTS) confirmed moderate radical scavenging activity, indicating that bioactivity is retained after cold-press extraction. These compositional and functional traits highlight the potential of the seed cake as a raw material for natural biopesticides, biofertilizers, and value-added agro-industrial products. However, due to its unusual fatty acid profile and possible anti-nutritional factors, feed applications should proceed with caution and be preceded by targeted safety evaluations.

## 1. Introduction

*Koelreuteria paniculata* Laxm., a saponaceous tree belonging to the family *Sapindaceae*, commonly known as the golden rain tree, is a deciduous ornamental species native to East Asia [1]. Due to its high aesthetic value, drought tolerance, and adaptability to nutrient-poor and polluted urban environments [2], it has been widely introduced into city landscapes across Europe, including Serbia. Previous studies by Ljubojević et al. have shown that this species demonstrates both ecological plasticity and significant biomass productivity in urban habitats [3]. Beyond its ornamental and ecosystem service roles, *K. paniculata* represents a multifunctional plant resource, yielding a wide range of biologically active compounds from both vegetative and generative organs. Earlier research has highlighted its potential as a feedstock for the food industry [4], the cosmetic and pharmaceutical sectors [5,6,7,8], and biodiesel production [9,10]. More recent developments have further underscored its untapped potential in sustainable applications. For instance, bract extracts have been successfully integrated into multifunctional green food packaging films in combination with titanium-doped carbon dots derived from bio-waste and a chitosan/locust bean gum matrix [11]. The stem bark and flower ethanol extracts of *K. paniculata* demonstrated notable antibacterial activity against *Bacillus subtilis*, *Bacillus cereus*, *Escherichia coli*, *Pseudomonas aeruginosa*, and *Proteus vulgaris* [6], while the seeds were effective against *Micrococcus tetragenus*, *Shigella dysenteriae*, and *E. coli* [12], suggesting the species as a promising source of bioactive compounds with antimicrobial potential. Furthermore, the seed ethanolic extract, as well as cold-pressed seed oil, demonstrated strong aphicidal effects against the woolly apple aphid (*Eriosoma lanigerum*), with mortality rates ranging between 86% and 100% [13]. The presence of phenolic acids (e.g., gallic, protocatechuic), flavonoids (quercetin and luteolin derivatives), and fatty acids suggests that the seed derivatives could serve as natural, plant-based biopesticides. In *K. paniculata*, a diverse array of bioactive compounds has been identified, highlighting its potential for biopesticide and pharmacological applications. Early studies reported the presence of gallate derivatives, cyanolipids, and flavonoids [14,15]. Later, Mostafa et al. [1] expanded this profile by isolating triterpenoidal saponins, including a tridesmosidic saponin named Paniculatosoid A and two monodesmosidic saponins, Paniculatosoid B and Paniculatosoid C, along with eleven additional known compounds such as luteolin derivatives, glycosyloxyflavones, and gallate derivatives.

Following oil extraction, the residual seed cake remains rich in bioactive secondary metabolites and essential nutrients. Rather than being discarded, this by-product can be valorized in accordance with circular-economy principles [16]. Previous research [17] has demonstrated that seed cakes, bark, and leaves from related species serve as potent sources of natural antioxidants. Although the primary focus in previous research was on oil- and extract-based products, the residual seed cake, often regarded as agricultural waste, remains underexplored despite retaining significant quantities of bioactive constituents and nutrients [18]. Investigating its potential aligns with zero-waste valorization pathways and contributes to a more holistic use of plant resources. There is a research gap investigating whether the *K. paniculata* seed cake may have biofertilizing potential or could be used as a component in animal nutrition. Although the literature on these aspects remains limited, the multifunctional utility of *K. paniculata* seed cake fits the strategic framework of the circular bioeconomy, transforming landscape-horticultural residues into value-added products that reduce environmental impact and improve sustainability in crop and livestock production.

However, to date, no study has systematically characterized the phytochemical profile of *K. paniculata* seed cake or evaluated its multifunctionality across pesticidal, fertilizing, and nutritional domains. Therefore, the aim of this study is to perform a comprehensive chemotyping of *K. paniculata* seed cake and evaluate its potential for use as a biopesticide, biofertilizer, and feed additive, instead of considering it as waste, thus contributing to sustainable and circular agricultural systems.

## 2. Results and Discussion

### 2.1. Basic Composition

**Figure 1:**
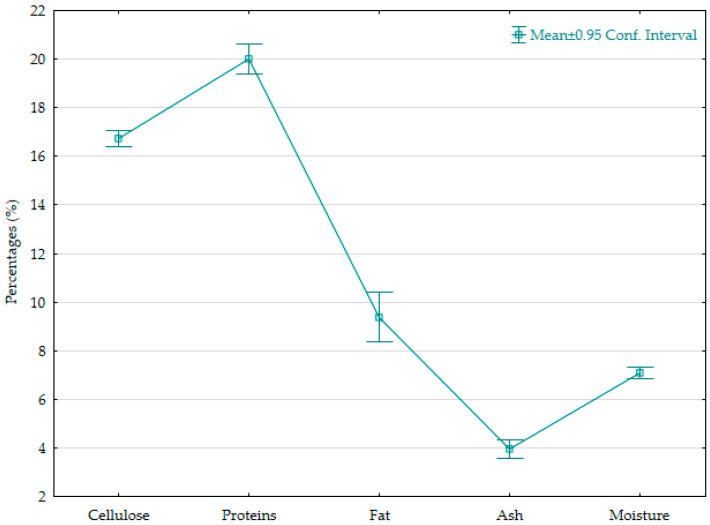
Basic composition of Koelreuteria paniculata seed cake (on a dry-matter basis).

Basic composition and chemical analyses of the seed cake, carried out in two replicates, showed very stable values between samples (Figure 1). The mean values were 16.72 ± 0.04% for cellulose, 20.01 ± 0.07% for protein, 9.39 ± 0.11% for fat, followed by 3.95 ± 0.04% ash and 7.08 ± 0.03% moisture in the sample. Moderate cellulose content can reduce digestibility in monogastric animals due to the limited enzymatic breakdown of structural carbohydrates, which is why dietary fiber was once considered an antinutrient. However, dietary fiber has recently gained increased attention for its functional value in improving gut health in monogastric animals [19]. The same fiber content can be beneficial for ruminants, in which dietary fiber plays a key role in maintaining rumen health and stimulating microbial activity, as it is known that dietary fiber can contribute nutritional value to animals, directly by providing energy and indirectly by improving gut health and immune function [20]. Fiber-rich agricultural by-products such as this have demonstrated potential to enhance animal performance and health when incorporated as functional feed additives, contributing to both nutritional value and gut-health modulation. Therefore, the seed cake residue may be particularly suited for ruminant diets or as part of integrated feed strategies aimed at sustainable livestock production [21].

The relatively high protein content indicates the potential of *K. paniculata* seed cake to be used as an alternative protein source in animal nutrition, especially when combined with its favorable amino acid profile, as previously surveyed for neem seed cake, another bioinsecticidal oil-providing species [22]. Both species are rich in saponins that may negatively affect protein digestibility and decrease feed intake due to their bitter taste and irritating effect on the throat [23]. However, saponins may interact with cholesterol in cell membranes, leading to membrane damage and cell rupture, and, in the rumen of ruminants, they can selectively eliminate protozoa, thereby improving nitrogen utilization efficiency and potentially enhancing animal productivity, as stated by Kholif [24]. Tannins are also known for reducing feed digestibility in both ruminants and non-ruminants by reacting with proteins, enzymes, or amino acids following enzymatic or non-enzymatic oxidation, resulting in the formation of various complexes [25,26]. Tannins in small amounts can have a positive effect in ruminant nutrition by reducing methanogenic fermentation in the rumen, thereby contributing to sustainable livestock production [27]. To ensure the safe use of *K. paniculata* seed cake in animal nutrition, the application of processing techniques for antinutrient removal is necessary [28]. Numerous studies suggest that heating and autoclaving are among the most efficient methods for reducing various antinutritional factors [28,29,30].

The fat content of *K. paniculata* seed cake (9.39%) is consistent with values reported in the literature for residual fat following mechanical pressing of oil-rich seeds [31,32]. As stated by Kokić et al. [33], oilseed processing by-products (meals and cakes) exhibit considerable variation in fat content (ranging from 1.1% to 16.3%), depending on the method of by-product generation. Oilseed meals are obtained through solvent extraction and therefore have a lower fat content compared to cakes produced by mechanical pressing, which retain a higher fat proportion and consequently contribute significantly to the energy value of animal diets.

Furthermore, the seed cake exhibited a relatively low moisture content of 7.08%, which is advantageous from both a storage- and application-perspective. Agricultural by-products are often rich in water, and high-fiber materials with elevated moisture levels are particularly prone to microbial spoilage. Such conditions can promote mold growth and the formation of toxic mycotoxins, which are known to impair animal performance and, in severe cases, cause mortality [34]. In this regard, the low moisture level of *K. paniculata* seed cake minimizes the risk of fungal contamination, thereby enhancing its safety and shelf life as a feed additive or soil amendment.

Possessing those properties, the seed cake also demonstrates biofertilizer potential. The protein and fat residues provide organic matter and slow-releasing nitrogen sources upon decomposition, while the cellulose fraction adds to soil structure and microbial activity [35]. The ash content may indicate useful macro- and micronutrients for plants. Such a composition is favorable for use in composting or direct soil amendment, especially in organic or low-input farming systems. However, further studies on the degradation rate, mineralization dynamics, and possible phytotoxicity would be required to confirm its agronomic efficiency and environmental safety.

Finally, following aqueous or ethanolic extraction of phenolic compounds that would be used as biopesticides, the majority of the seed cake’s core determined constituents (proteins, lipids, cellulose, and ash) remain largely preserved in the residual biomass. Cellulose, being insoluble in both water and ethanol, retains its structural integrity and continues to provide fiber beneficial for ruminant nutrition or soil conditioning upon the microorganisms’ actions [36]. Proteins in the seed cake would only be marginally affected by aqueous extraction, with minor losses of soluble fractions, while ethanolic extraction exerts minimal influence [37,38]. Lipid content may slightly decrease due to partial solubility in ethanol, whereas mineral components (ash) remain stable in both cases, preserving their agronomic relevance. Accordingly, the defatted and dephenolized residue might retain its value as a fiber- and protein-rich byproduct, particularly suitable for ruminant feed or organic soil amendment.

### 2.2. Amino Acid Content

Results related to the amino acid composition of the analyzed sample, expressed as grams per 100 g of protein, are presented in Table 1. Glutamic acid was the most abundant amino acid, accounting for 18.2 ± 0.65 g/100 g protein, followed by arginine and aspartic acid (12.1 ± 1.11 and 10.9 ± 0.16 g/100 g protein, respectively) and glycine (5.92 ± 0.20 g/100 g protein), as well as lysine, serine, and leucine (5.52 ± 0.33, 5.06 ± 0.07, and 5.01 ± 0.54 g/100 g protein, respectively). Essential amino acids such as valine, phenylalanine, alanine, and threonine were present in moderate amounts (4.91 ± 0.65 down to 3.94 ± 0.16 g/100 g protein). Isoleucine, proline, tyrosine, and histidine were detected in smaller amounts (2.86 ± 0.42 down to 2.04 ± 0.09 g/100 g protein), whereas cystine and methionine were the least represented (0.68 ± 0.08 and 0.43 ± 0.22 g/100 g protein). The total quantified amino acids amounted to 90.25 g/100 g of protein. The presence of a balanced profile of essential and non-essential amino acids, along with high overall amino acid content, suggests that the seed cake may have promising potential as a protein-rich component in animal feed formulations, as previously demonstrated by Tomičić et al. [39] for soybean meal, soybean cake, and sunflower meal. However, the low methionine content may require supplementation depending on the nutritional needs of the target species [40,41,42] or inclusion of the *K. paniculata* seed cake into specialized mixtures. On the other hand, lysine, an important food and feed component [43], was moderately abundant in the present study.

Regarding the possible organic-fertilizer application of seed cake, amino acids certainly aid its role in nutrient provision. Namely, previous studies have shown that amino acid-based biofertilizers represent a promising group of natural substances used to enhance plant growth and crop yields [67]. They consist of free amino acids or short peptides, which are readily available to plants and can directly participate in their metabolism. Furthermore, amino acid-based biofertilizers often help plants overcome stressful conditions, such as drought or high temperatures, by improving their resilience and vitality. Given the high protein content and amino acid richness of the investigated seed cake, there is a possibility of producing free amino acids and short peptides through chemical or enzymatic hydrolysis, as shown for dairy wastes [68], enhancing soil health, increasing crop yields, and reducing dependence on synthetic inputs.

Regarding the bioactive potential, certain amino acids, like aspartic acid, glutamic acid, cystine, arginine, valine, and phenylalanine, can express antimicrobial activity, either directly or through their influence on microbial metabolism and the environment. It was previously noted that pesticides produced from higher plants and algae contain biologically derived compounds, including a variety of chemical classes, such as terpenes, alkaloids, quinones, aldehydes, saccharides, and flavonoids, as well as amino acids [69]. Amino acid presence in protein-rich extracts may enhance antimicrobial potential, particularly in synergy with phenolic compounds or fatty acids [44,46,48,50,52,53,55,57,59,61,63,65]. The seed cake investigated in this study was rich in glutamic acid, arginine, and aspartic acid, followed by glycine, lysine, serine, and leucine. It was the incorporation of glutamic acid into chitosan–phthalocyanine systems that enhanced their antimicrobial properties, highlighting the role of amino acids as bioactive components and providing a rationale for examining their contribution to biopesticide development in this study [46]. The incorporation of the second-most abundant amino acid—arginine—was previously demonstrated to increase the antimicrobial nanozyme activity by nearly 1000-fold, primarily through enhanced ROS generation and disruption of bacterial membranes. These findings underline the potential of L-arginine as a key bioactive amino acid for boosting antimicrobial efficacy [65]. As for the third-ranked aspartic acid, Monteiro et al. [44] identified it as an effective antibiofilm agent against *Pseudomonas aeruginosa*, a pathogen affecting not only humans but also animals and plants. By interfering with bacterial adhesion, aspartic acid suppresses the pathogen, while, in combination with tobramycin, it significantly enhances antimicrobial efficacy, positioning this acid as a valuable bioactive adjuvant.

Additionally, some amino acids (notably arginine, glutamic acid, glycine, and phenylalanine) can act as insect-feeding deterrents or disruptors of development, particularly when concentrated or imbalanced [45,47,49,51,54,56,58,60,62,64,66]. This makes them potential contributors to bioactivity in protein-rich extracts used in biopesticide development. In developing a nanobiopesticide, Chen et al. [47] revealed that aspartic acid enhanced the insecticidal efficacy of spinosad and implied the broader potential of amino acids, especially glutamic acid, as key elements in designing environmentally friendly biopesticides. Similarly, a recent study proved that external application of phenylalanine contributes to the synthesis of volatile compounds that repel *Tuta absoluta* in tomato [64]. Another major pest, the cotton aphid *Aphis gossypii*, reduces cotton yield through sap feeding and virus transmission, for which Yousaf et al. [66] developed transgenic cotton lines using plant-mediated RNA interference (RNAi) targeting arginine kinase (AK)**,** an essential enzyme for insect energy metabolism. In fact, numerous naturally occurring amino acid derivatives exhibit notable bioactivity against weeds, fungi, and insect pests, of which several have been successfully commercialized and are currently employed as crop protection agents [70].

Furthermore, amino acid-based biosurfactants, derived from renewable sources, represent a promising eco-friendly alternative to conventional chemical surfactants [71]. Due to their biodegradability, low toxicity, and stability across a range of temperatures and pH levels, they are especially suitable for use in agriculture. The investigated seed cake was especially abundant in glutamic and aspartic acid, as well as arginine, lysine, and glycine, which is particularly interesting given that the cultivation of *Burkholderia* AD24 in the presence of glycine, glutamine, arginine, and lysine, in the most recent study, was shown to stimulate the production of fungistatic volatiles that suppressed the growth of *Rhizoctonia solani* [72]. In another study, antimicrobial hydrogels that were based on the commercial phenylalanine and leucine exhibited selective bactericidal activity against Gram-positive bacteria, indicating their potential as antimicrobial coatings [73]. On the other hand, alanine derived from *Mirabilis jalapa* demonstrated potential as a biopesticide by targeting insect glutamate receptors, disrupting neuromuscular signaling and immune responses, and ultimately leading to insect mortality through sustained interference with signal transduction [74].

### 2.3. Fatty Acid Content

The fatty acid composition of the sample, expressed as grams per 100 g of total fatty acids, along with noted antimicrobial and insecticidal activities reported in the literature, is shown in Table 2. Saturated fatty acids (SFA) were present in relatively low amounts (10.1%), with palmitic acid (6.3%) being the predominant SFA. Monounsaturated fatty acids (MUFA) represented the major fraction of the total fatty acids (75.8%), dominated by eicosenoic acid (43.0%) and oleic acid (31.1%), while erucic acid was detected in a minor proportion (1.7%). Polyunsaturated fatty acids (PUFA) accounted for 13.7%, with linoleic acid (12.8%) as the principal PUFA. Several of the identified fatty acids, particularly eicosenoic, linoleic, oleic, erucic, and palmitic acids, have been previously reported to possess both antimicrobial and insecticidal properties, suggesting potential bioactive roles in plant defense or biopesticide formulations [13,75,76,77,78,79,80,81,82,83,84,85,86,87].

In previous studies, GC/MS analysis revealed that date palm seeds are particularly rich in oleic and lauric acids, and their aqueous extract exhibited fungitoxic activity, indicating potential for antimicrobial applications [78], while palmitic and lauric acids derived from palm kernel oil demonstrated effective antibacterial activity against *Staphylococcus aureus* and *E. coli* [75]. At the same time, palmitic acid derived from *Jatropha curcas* possessed an insecticidal effect on armyworms of the genus *Spodoptera* [76], indicating a simultaneous dual bioactive effect. Investigating Damask rose, Ghavam et al. [79] reported that, besides volatile compounds highly present in its oil, another compound—oleic acid, also significantly present in the investigated seed cake—plays a crucial role in the antibacterial and antifungal effects. Interestingly, another species from the *Sapindaceae* family *Paullinia pinnata* L., rich in fatty acids, exhibited significant antibacterial activity against both Gram-positive and Gram-negative bacteria. In this case, palmitic, oleic, eicosanoic, and stearic acids weakly to moderately affected *S. aureus*, *B. subtilis*, *E. coli*, and *P. aeruginosa* [83]. Fatty acids detected in *K. paniculata* seed cake in the present study, particularly palmitic, oleic, eicosenoic, and linoleic, have also been identified in *Ulva rigida* extracts, where they exhibit marked antibacterial and antifungal effects [84]. On the other hand, comparable research has highlighted oleic acid as the principal contributor to the biopesticidal efficacy of cassava seed oil, achieving more than 90% larval mortality in *Spilarctia obliqua* and effectively controlling *Aphis craccivora* populations [88]. Similarly, moringa seed oil (rich in arachidic, behenic, oleic, and palmitic acids) has demonstrated potent activity against *Tetranychus urticae* (Koch) and the cotton mealybug *Phenacoccus solenopsis* [89].

Eicosenoic acid, characteristic of and identified as the most abundant fatty acid in *K. paniculata* seed cake, is present in many plant oils and nuts, with particularly high levels in jojoba oil, where it accounts for about 70% of the total fatty acids. It has been increasingly recognized for its potential health-promoting properties. Previous studies have shown that eicosenoic acid can protect pancreatic β-cells from lipotoxicity, enhance insulin secretion, and work synergistically with omega-3 fatty acids to attenuate excessive lipid accumulation in hepatocytes [90]. Moreover, emerging evidence indicates that eicosenoic (gondoic) acid may contribute to neurodevelopment [90], while other research has demonstrated its anti-inflammatory activity [91].

A less abundant MUFA in *K. paniculata* cake was erucic acid, detected at 1.7% of total fatty acids. Although present in relatively low concentrations, erucic acid is generally considered undesirable in both animal and human diets due to its association with myocardial lipidosis and other pathological cardiac lesions when consumed in excessive amounts [92]. Nevertheless, erucic acid is regarded as safe when ingested within reasonable dietary limits. Regulatory guidelines set by the European Commission (2006) [93] stipulate a maximum allowable level of 20 g/kg of total fatty acids in vegetable oils and fats placed on the market for the final consumer or for use as an ingredient in food. In the present study, the erucic acid concentration measured in *Koelreuteria paniculata* cake was below this regulatory threshold, indicating no safety concerns regarding erucic acid content. Despite the historical concerns about its toxicity at high levels, erucic acid also exhibits several beneficial biological properties, including antibacterial, antiviral, anti-inflammatory, anticancer, cytotoxic, and neuroprotective activities [94].

From a nutritional standpoint, the high proportion of unsaturated fatty acids further supports the feed potential of the sample. As these fatty acids play important roles in animal growth, reproduction, and immune function, *K. paniculata* seed cake could be considered a potentially valuable source of dietary lipids in feed applications, with one essential precaution. Namely, according to the fatty acid profile, the *Koelreuteria* seed cake is very unusual compared to standard feed cakes, especially with its extremely high eicosenoic acid (C20:1n9) amounting to 43%. Since all major fatty acids here have documented antimicrobial and insecticidal effects, these fatty acids could suppress beneficial gut flora or affect rumen fermentation. While the antimicrobial property is the greatest value of *Koelreuteria* oil for biopesticide applications, it may have minor, potentially negative effects in some animals, highlighting the need for further research and optimization through performance and safety trials.

### 2.4. Composition of Individual Polyphenols

To further fortify the theses on the *K. paniculata* seed cake’s potential as a feed additive, biopesticide, or even biofertilizer, detailed analyses of its phenolic composition were undertaken. *K. paniculata* is exceptionally rich in bioactive compounds, among which the most notable are polyphenols (including flavonoids and tannins), saponins, fatty acids, as well as previously mentioned specific triterpenoids and cyanolipids. The obtained compositional profile of *K. paniculata* seed cake confirmed its potential as a biopesticide, biofertilizer, or feed additive. The most abundant compound was ellagic acid (945.27 ± 16.98 μg/g), followed by rutin (903.68 ± 19.21 μg/g), catechin (791.44 ± 13.24 μg/g), and gallic acid (749.32 ± 12.22 μg/g), while quercitrin and quercetin were detected in very low concentrations (14.28 ± 0.33 μg/g and 5.11 ± 0.11 μg/g, respectively) as shown in Table 3.

Phenolics are widely acknowledged for their strong antioxidant properties and their roles in mitigating oxidative stress. In particular, catechin has been shown to enhance systemic antioxidant capacity, stimulate lipid metabolism, and reduce low-grade inflammation associated with adiposity in mice [95]. The presence of such compounds further supports the nutritional and functional value of the seed cake, not only in terms of its basic composition (fiber, protein, fat), but also as a source of health-promoting phytochemicals. The presence of quercitrin and quercetin, although in very low concentrations, contributes to the overall bioactive profile, highlighting the multifunctional potential of the seed cake in sustainable agricultural applications. The predominance of ellagic acid, rutin, and catechin indicates a phenolic profile rich in flavonoids and building blocks for hydrolyzable tannins, contributing to antioxidant, antimicrobial, and potential anti-inflammatory properties [21]. However, the impact on feed acceptability requires attention, as ellagic and gallic acids (hydrolyzable tannins) may reduce protein digestibility by forming complexes with proteins and enzymes. Catechin, a building block for condensed tannin, could also affect palatability at higher levels. Despite these concerns, the sample’s moderate tannin content and trace levels of quercetin derivatives suggest no significant negative impact on feed potential [96], especially with ethanolic extraction mitigating antinutritional effects.

Importantly, many of these phenolics also exhibit notable antimicrobial and insecticidal properties. Ellagic acid has been reported to inhibit the growth of several bacterial, viral, and fungal pathogens [97,98] by disrupting cell membranes and interfering with microbial enzymes [99], with concomitant insecticidal activity against aphids [100], moths [101], and mosquitoes [102]. Additionally, gallic acid demonstrates both bactericidal and fungicidal activity, often linked to its ability to induce oxidative stress in microbial cells. The phytochemical profile of our sample, characterized by a gallic acid concentration of 107 μg/mL, significantly exceeds that of the *Mangifera indica* peel methanolic extract (55.60 μg/mL), reported by Albrahim et al. [103] while investigating antimicrobial, anticancer, and antiviral efficacy. Findings of Punia et al. [104] suggest that gallic acid, even at lower concentrations (LC30), can impair the growth of *Spodoptera litura* larvae without causing significant harm to its parasitoid *Bracon hebetor*, indicating its strong potential for biopesticide applications. Containing albeit 14.45 µg/mL of gallic acid (fortified by other phenolic acids), methanol extract from *Pluchea dioscoridis* was effective against the nymphal instar of *Aphis gossypii* Glover and adult females of *Phenacoccus solenopsis* Tinsley after 24 and 48 h of treatment [105].

Catechin [106] and rutin [107], both in significant amounts present in the investigated seed cake, have shown insecticidal activity against various pests through mechanisms such as feeding deterrence, inhibition of digestive enzymes, and oxidative damage. Additionally, rutin and quercetin have been implicated in plant defense signaling and oviposition deterrence [108]. Considering its antimicrobial potential, rutin (abundant in the investigated seed cake), its methylated derivatives, and a novel flavonol glycoside isolated from *Ruta chalepensis* exhibited antimicrobial activity against four Gram-positive and Gram-negative bacterial strains, as well as *Candida albicans*, as demonstrated by Al-Majmaie et al. [109]. The antibacterial properties of rutin against various bacterial strains have been well-documented. Notably, rutin exhibits significant growth-inhibitory effects on *E. coli*, as well as on *Proteus vulgaris*, *Shigella sonnei*, and *Klebsiella* species, particularly when assessed in honey matrices, as well as food systems [110,111,112]. Additionally, rutin demonstrates antimicrobial activity against *Pseudomonas aeruginosa* and *Bacillus subtilis*. A study by Goda et al. [113] showed that the major antimicrobial compounds in licorice extract were exactly ellagic acid, quercetin, rutin, and catechin. The extract in their study exhibited substantial antibacterial action, effectively suppressing the development of all tested harmful bacteria, such as *B. subtilis*, *Staphylococcus aureus*, and *P. aeruginosa*.

Furthermore, when applied to soil, phenolic compounds from seed cake may contribute to rhizosphere modulation by suppressing nematodes [114], soil-borne pathogens such as *Rhizoctonia solani*, and stimulating beneficial microbes, thereby supporting the biofertilizer potential of the material. In a previous study, it was noted that phenolic-rich olive pomace can serve as a biofertilizer-compost due to the high organic content and minerals [115].

### 2.5. Antioxidative Potential

**Figure 2:**
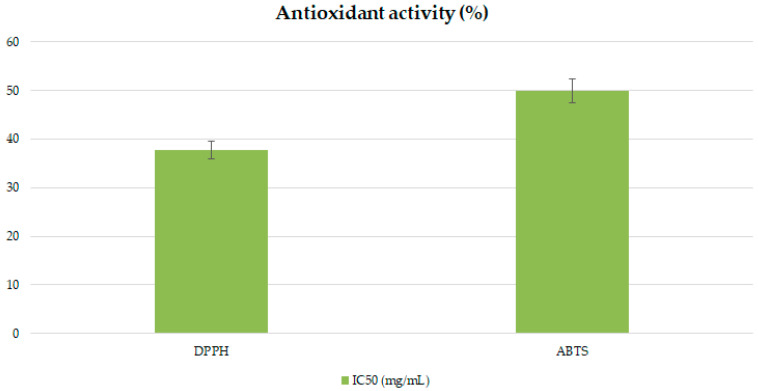
Antioxidant activity (%) of *Koelreuteria paniculata* seed cake extract determined by DPPH and ABTS radical scavenging assays.

The antioxidant potential of the extract obtained from the cold-pressed seed cake of *K. paniculata* was evaluated using both DPPH and ABTS radical scavenging assays (Figure 2). In the DPPH assay, the extract achieved up to 81.53% inhibition, with an IC_50_ value of 37.79 mg/mL, indicating a moderate antioxidant capacity. In the ABTS assay, the highest inhibition was 85.58%, with an IC_50_ value of 49.88 mg/mL. Although the activity was lower compared to standards such as fruit-derived ascorbic acid [116], the results demonstrate that the cold-pressing residue retains bioactive properties, supporting its potential application in the formulation of natural biopesticides or as a value-added by-product in agro-industrial processes.

The slightly higher IC_50_ in the ABTS assay compared to DPPH can be attributed to differences in radical reactivity and solubility, as ABTS^+^• is more hydrophilic and interacts with a broader spectrum of compounds, which may influence the apparent antioxidant efficiency.

Regardless, both assays confirmed the appreciable antioxidant potential of the extract, significant in animal feed, bio-preservation, or organic amendments. Seed cake extracts are known to contain phenolic acids and flavonoids with strong antioxidant activity, including gallic acid and quercetin derivatives. Similar findings have been reported for seed cakes of other oil-producing species, such as *Nigella sativa* and *Moringa oleifera*, where antioxidant activity strongly correlates with extractability and the residual phenolic content after oil extraction [117,118]. Although the IC_50_ values obtained here are higher than those of pure synthetic antioxidants like BHT (typically below 0.1 mg/mL), the extracts still demonstrate promising natural antioxidant potential. This is particularly relevant for valorizing agro-industrial by-products, providing bioactive input for the development of low-cost biopesticides, feed additives, or biostimulants. Moreover, the observed antioxidant activity could enhance the stability and effectiveness of formulations designed to mitigate oxidative stress in pest-infested or drought-stressed crops.

### 2.6. Correlation Analyses Within and Between Chemical and Phytochemical Parameters and Antioxydant Activity

Aiming to understand interactions within the same group of chemicals as well as between investigated parameters and obtained results for antioxidant activity, a correlation analyses were performed. Due to the investigation of two independent technical samples, as well as very uniform results reflected in the small standard deviations, even upon filtering out parameters with absolutely negligible variance, the majority of computed correlation coefficients remained numerically almost identical. However, several interesting correlations arose as statistically significant and useful as markers in future investigations. Namely, among amino acids, threonine and proline were statistically significant and negatively correlated with r = −0.80, while serine and lysine showed a statistically significant positive relation (r = 0.75). However, on a broader reviewed sample, Kumar et al. [119] reported that Pearson’s correlation analysis among different amino acids showed no negative correlations between the amino acids in plants. Within the fatty acids, palmitic acid was positively correlated with arachidic acid (r = 0.77) and stearic with linoleic (r = 0.65), while, due to its belonging to monounasutared fatty acids, eicosenoic acid expressed a strong correlation with MUFA, reaching r = 0.76. Interestingly, Andersen and Gorbet [120] noted that oleic acid was strongly positively correlated solely to eicosenoic acid, which were both the most represented in the investigated seed cake. Furthermore, within phenolic compounds, quercitrin levels were negatively related to rutin amounts (r = −0.66), representing the only statistically significant correlation for this group of chemicals. This relation is expected, since rutin and quercitrin are both forms of quercetin—an increase in one compound often occurs at the expense of the other.

**Figure 3:**
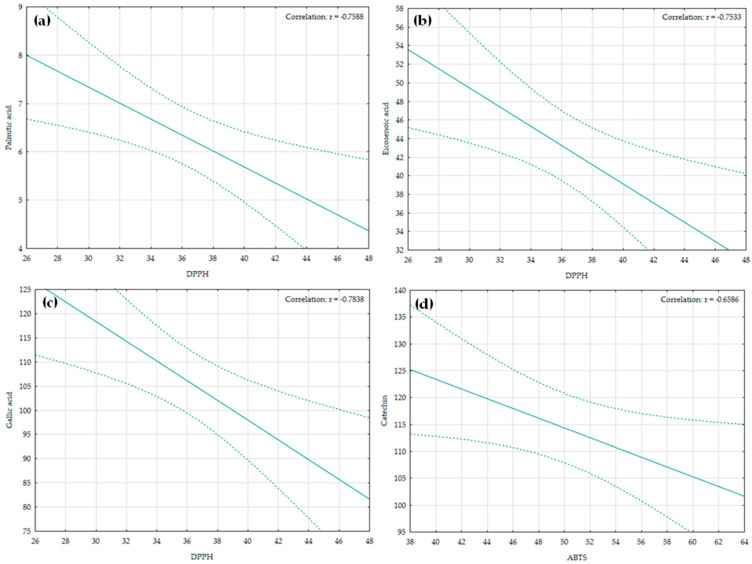
Statistically significant correlations of the investigated *K. paniculata* phytocemical parameters and DPPH/ABTS radical scavenging assays. (**a**) Correlation analysis between palmitic acid and DPPH radical scavenging activity; (**b**) Correlation analysis between eicosenoic acid and DPPH radical scavenging activity; (**c**) Correlation analysis between gallic acid and DPPH radical scavenging activity; (**d**) Correlation analysis between catechin and ABTS radical scavenging activity.

When observed with antioxidant tests—DPPH and ABTS—four correlations arose as significant. As presented in Figure 3a–c, increases in palmitic, eicosenoic, and gallic acid contents were strongly correlated with the lowering of the DPPH activity threshold, indicating that higher concentrations of these compounds would require lower DPPH levels to achieve inhibition. Previously, Malinda et al. [121] determined that gallic and ellagic acid exhibited DPPH radical scavenging activity with IC_50_ values of 13.2 and 15.9 μM, suggesting slightly better DPPH radical scavenging activity associated with gallic rather than ellagic acid. Similarly, Corradi et al. [122] noted the close relationship between gallic acid and antioxidant activity. In contrast, ABTS activity was significantly correlated with catechin content, showing that higher catechin levels might have enhanced the ABTS radical scavenging capacity (Figure 3d). Nevertheless, there is a high correlation between antioxidant assays and polyphenol (catechin and caffeine) contents [123], reducing the number of analyses to be performed when assessing the bioactive potential.

## 3. Materials and Methods

### 3.1. Seed Cake Harnessing

**Figure 4:**
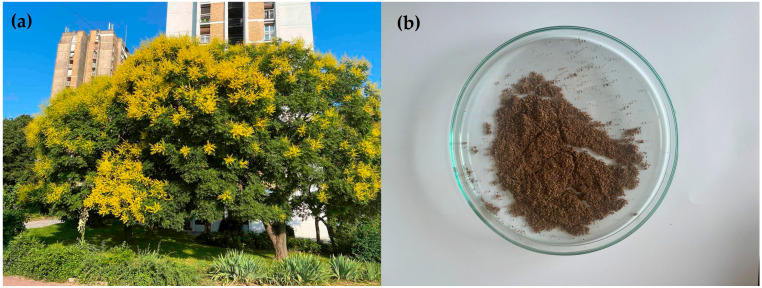
Mature amenity *K. paniculata* tree (**a**), which served as a source of seed cake (**b**).

The seed cake used in this research was a bio-waste derived from research focused on *K. paniculata* oil [13]. Briefly, mature seeds of *K. paniculata* were collected in October 2022 and 2023 from a single healthy and well-developed ornamental tree cultivated under urban continental-climate conditions in Novi Sad, Serbia (Figure 4a). The selected specimen, approximately 7 m in height and canopy width, was free of visible damage or decline and previously characterized in biodiesel-related and biopesticide studies [3,10,13]. Seed harvesting was performed from all four canopy orientations to ensure representative sampling, resulting in a composite bulk of 2 kg.

After collection, seeds were air-dried at room temperature for seven days, reaching a stable moisture level approximately 6–8%, a level considered optimal for safe storage and efficient oil pressing [124,125]. Initially, seeds were considered dried once the seed coats ceased to lose water, which was evident from their black-gray coloration, as opposed to a glossy black appearance, later confirmed by moisture determination under laboratory conditions. Mechanical cold pressing using the screw press, explained and illustrated in detail by Tomić et al. [10], was then conducted without prior thermal or chemical treatment, yielding crude oil as the primary product. The residual seed cake, obtained post-pressing, was carefully collected and stored in airtight containers under dry conditions to prevent degradation of bioactive compounds. This defatted seed cake, considered a by-product of oil extraction, served as the material for subsequent chemical characterization and assessment of its biopesticidal, biofertilizing, and feed potential. Due to the stable values obtained in the first two replicates, all analyses except HPLC were performed on two finely ground seed cake samples, which were considered technical samples (Figure 4b).

### 3.2. Basic Chemical Composition and Amino Acid Content

*K. paniculata* seed cake was analyzed for moisture, crude protein, crude ash, crude fat, and crude fiber. The moisture content was determined according to ISO method 6496:1999 [126], where the loss in mass of a portion of the sample was measured after drying at 103 °C ± 2 °C for 4 h ± 0.1 h, until a constant weight was reached. Crude protein content was determined by the Kjeldahl method according to ISO method 5983-1 [127], crude ash according to ISO method 5984 [128], crude fat according to ISO method 6492 [129], and crude fiber according to ISO method 6865 [130].

Amino acid analyses of the tested samples were performed by ion exchange chromatography using an Automatic Amino Acid Analyzer Biochrom 30+ (Biochrom, Cambridge, UK), according to Tomičić et al. [39]. The sodium-accelerated buffer system consisted of a set of four buffers with pH varying between 3.2 and 9.2 and a sodium hydroxide regeneration solution, with a flow rate of 35 mL/h. The technique was based on amino acid separation using strong cation exchange chromatography, followed by the ninhydrin color reaction and photometric detection at 570 nm, except for proline, which was detected at 440 nm. Samples were previously hydrolyzed in 6M HCl (Merck, Darmstadt, Germany) and, for the determination of tryptophan, in 6M NaOH at 110 °C for 24 h. After hydrolysis, samples were cooled to room temperature and dissolved in 25 mL of loading buffer (pH 2.2) (Biochrom, Cambridge, UK). Subsequently, prepared samples were filtered through a 0.22-μm pore size PTFE filter (Plano, TX, USA), and the filtrate was transferred into a vial (Agilent Technologies, Santa Clara, CA, USA) and stored in a refrigerator prior to analysis. The amino acid peaks were identified by comparison of retention times with retention times of an amino acid standard purchased from Sigma Aldrich (Amino Acid Standard Solution (Sigma-Aldrich, St. Louis, MI, USA)). The results were expressed as the mass of amino acid (g) in 100 g of protein.

### 3.3. Fatty Acid Content Analysis

Lipids from the cake were extracted with a chloroform/methanol mixture (2:1) for 2.5 h. Then, the lipids were converted to fatty acid methyl esters (FAME) with 14% boron tri-fluoride in methanol solution according to the SRPS EN ISO 12966-2:2017 method [131]. The profile of FAME was determined using an Agilent 7890A gas chromatograph (Agilent Technologies, Santa Clara, CA, USA) fitted with a fused-silica capillary column SP-2560 (100 m × 0.25 mm, d = 0.20 μm; Supelco, Bellefonte, PA, USA) and equipped with a flame ionization detector (FID). The injection volume was 1 μL, and the split ratio was 1:30. Helium was used as a carrier gas. An initial column temperature of 140 °C was maintained for 3 min, followed by an increase to 220 °C at a rate of 3 °C/min and holding it for 5 min. Finally, the column temperature was increased to 240 °C at a rate of 2 °C/min and was held constant for 10 min. The detector and injector temperatures were set at 250 °C. The identification of FAME was conducted by comparing their retention times with those of an authentic standard (Supelco 37 Component FAME Mix; Sigma-Aldrich, St. Louis, MI, USA). The content of individual FAME was expressed as a percentage of the total identified FAME.

### 3.4. HPLC Analysis

Ethanolic extract preparation included a simple methodology so that, if proven successful, could be repeated anytime and anywhere, regardless of the laboratory conditions. Dried seed cake (50 g) was extracted with 97% ethanol (500 mL) as a solvent by simply soaking for four weeks at room temperature. As a standard protocol, the analysis of the ethanolic extract of seed cake was carried out in triplicate using an Agilent 1260 RR high-performance liquid chromatography (HPLC) system (Agilent Technologies, Waldbronn, Germany) fitted with a diode-array detector operating over a wavelength range of 190 to 550 nm. Separation of compounds was achieved using a Zorbax SB-C18 reversed-phase analytical column (Agilent) with a length of 150 mm, an internal diameter of 4.6 mm, and a particle size of 5 μm. The mobile phase consisted of two solvents: Solution A was 1% (*v*/*v*) orthophosphoric acid in water, and solution B was acetonitrile. A gradient elution was applied as follows: 90–85% A from 0 to 2.6 min; maintained at 85% A from 2.6 to 8 min; then 85–80% A from 8 to 10.8 min; held at 80% A until 18 min; then decreased to 70% A by 23 min; 50% A by 25 min; 30% A by 27 min; 10% A by 29 min; and finally reaching 0% A by 31 min, which was held constant until 34 min. Detection was performed at 260, 280, 320, and 360 nm. The flow rate was maintained at 0.8 mL/min, with an injection volume of 8 μL, and the column temperature was set to 40 °C. Compounds were identified by matching their retention times and UV spectra to those of known standards. The following standard compounds were used: gallic acid, ellagic acid, rutin, catechin, quercetin, and quercitrin, which were purchased from Sigma-Aldrich (Darmstadt, Germany). Quantitative analysis was based on calibration curves, and results were expressed in micrograms per g (μg/g). The applied HPLC method was found to be linear within five different concentrations for each of the four analyzed compounds. The correlation coefficients (R^2^) for the quantified standards were close to 1 (R^2^ > 0.998), indicating strong linearity. The limits of detection (LOD) and quantification (LOQ) for the four analyzed compounds are summarized in Table 4. The relative standard deviation (RSD) values were all below 2%, demonstrating the repeatability of the method.

### 3.5. Antioxidative Activity

#### 3.5.1. DPPH Radical Scavenging Assay

Antiradical activity of digestives by DPPH free radicals (DPPH Radical Scavenging Activity) was determined using a spectrophotometric method, which is based on the principle of measuring changes in the concentration of stable DPPH radicals in the presence of the test samples. This examination was performed according to the method of Morales and Jiménez-Pérez [132]. The solution of DPPH radicals, with a concentration 74 mg/mL, was prepared in ethanol (prepared on a daily basis). A total of 1000 μL of solution was mixed with 200 μL of the sample and placed in a dark place for 30 min at room temperature. At the same time, for each sample, a blank solution was prepared with water or the relevant buffer instead of the sample. After the reaction was completed, the samples were centrifuged at 14,500 rpm (Eppendorf Mini spin plus) for 5 min. The absorbance of the supernatants was measured at 520 nm (T80/T80+ UV-Vis Spectrophotometer, PG instruments Ltd., Leicestershire, UK). Antiradical activities of the samples were calculated according to the following equation:

$$Antioxidant\;\;activity\left(\%\right)=\frac{\left({Abs}_{blank}-{Abs}_{sample}\right)}{{Abs}_{blank}}\times 100$$

where Abs_blank_ is the absorbance of the blank solution, and Abs_sample_ is the absorbance of the sample solution.

#### 3.5.2. ABTS Radical Scavenging Activity Assay

This assay was specified by the ABTS radical cation decolorization assay as defined by Re et al. [133]. Briefly, 30 µL of protein sample was added to 3 mL of diluted ABTS radical cation solution (A_734nm_ = 0.7 ± 0.02), and the absorbance was measured after 10 min at 734 nm (T80/T80+ UV-Vis Spectrophotometer, PG instruments Ltd., Leicestershire, UK). Appropriate solvent blanks were run in each assay. The ABTS antioxidant activity was calculated as follows:

(1) $$Antioxidant\;\;activity\;\%=\frac{\left(A_{control}-A_{sample}\right)}{A_{control}}\times 100$$

where A_control_ is the concentration of ABTS in the blank (in the presence of buffer instead of protein extract), and A_sample_ is the concentration of ABTS in the sample (in the presence of protein extract) after 10 min of reaction.

### 3.6. Statistical Analyses

The results were processed with the program STATISTICA 13 (TIBCO Software Inc., 2017, Palo Alto, CA, USA), yielding basic descriptive statistics for the investigated parameters. Correlation analysis was performed using Pearson’s correlation coefficients, with statistically significant values determined at *p* < 0.05.

## 4. Conclusions

This study provided an insight into *Koelreuteria paniculata* seed cake chemical constituents, confirming its compositionally rich profile and potential valorization pathways within sustainable and circular agricultural systems. Proximate analysis revealed a balanced nutrient composition of proteins, fat, and cellulose, suggesting a role as a functional feed additive or soil amendment rather than a bulk protein source. The amino acid profile was dominated by glutamic acid, arginine, and aspartic acid, alongside a range of essential amino acids, while the fatty acid spectrum was notable for its unusually high proportion of monounsaturated fatty acids, particularly eicosenoic and oleic acids, with documented bioactive properties. In addition to macronutrient and lipid composition, the seed cake retained significant levels of phenolic compounds, with ellagic acid, rutin, catechin, and gallic acid as the major constituents, also proven for their bioactive potential. Additionally, antioxidant activity assays (DPPH and ABTS) demonstrated moderate radical scavenging capacity, confirming that bioactivity is preserved even after cold-press oil extraction. This combination of bioactive fatty acids and phenolics, in correlation with DPPH and ABTS levels, supports the seed cake’s potential application in natural biopesticide formulations, as well as in biofertilizer blends enriched with antimicrobial properties. While the compositional and obtained data suggest promising opportunities for high-value utilization of *K. paniculata* seed cake, caution is warranted regarding direct feed applications due to the unusual fatty acid profile and potential anti-nutritional factors typical of the Sapindaceae family. Future work should focus on targeted safety evaluations, detoxification strategies, and pilot-scale product development to translate these findings into practical, economically viable, and environmentally sound agro-industrial applications.

## Figures and Tables

**Table 1:** Amino acid content in the investigated *K. paniculata* seed cake, with a literature overview of both the direct and indirect roles in antimicrobial and insecticidal effects.

Amino Acid	g/100 g of Protein	Antimicrobial Effect	Insecticidal Effect
Aspartic acid	10.9 ± 0.16	✓ [44]	✓ [45]
Threonine	3.94 ± 0.16	/	/
Serine	5.06 ± 0.07	/	/
Glutamic acid	18.2 ± 0.65	✓ [46]	✓ [47]
Proline	2.17 ± 0.40	✓ [48]	✓ [49]
Glycine	5.92 ± 0.20	✓ [50]	✓ [51]
Alanine	4.08 ± 0.28	✓ [52]	/
Cystine	0.68 ± 0.08	✓ [53]	✓ [54]
Valine	4.91 ± 0.65	✓ [55]	✓ [56]
Methionine	0.43 ± 0.22	✓ [57]	✓ [58]
Isoleucine	2.86 ± 0.42	✓ [59]	✓ [60]
Leucine	5.01 ± 0.54	✓ [61]	✓ [62]
Tyrosine	2.16 ± 0.20	/	/
Phenylalanine	4.15 ± 0.40	✓ [63]	✓ [64]
Histidine	2.04 ± 0.09	/	/
Lysine	5.52 ± 0.33	/	/
Arginine	12.1 ± 1.11	✓ [65]	✓ [66]
Total amino acids (TAA)	90.3 ± 5.63		

**Table 2:** Fatty acid content in the investigated *K. paniculata* seed cake, with a literature overview of both the direct and indirect roles in antimicrobial and insecticidal effects.

Fatty Acid	Content g/100 g Total FA	Antimicrobial Effect	Insecticidal Effect
Palmitic C16:0	6.30 ± 0.04	✓ [75]	✓ [76]
Stearic C18:0	1.20 ± 0.06	✓ [77]	/
Oleic C18:1n9c	31.1 ± 0.57	✓ [78,79]	✓ [76]
Linoleic C18:2n6c	12.8 ± 0.42	✓ [80]	✓ [81]
Arachidic C20:0	2.60 ± 0.14	/	✓ [82]
Eicosenoic C20:1n9	43.0 ± 0.85	✓ [83,84]	✓ [13]
Eicosadienoic C20:2n6	0.90 ± 0.00	/	✓ [85]
Erucic C22:1n9	1.70 ± 0.07	✓ [86]	✓ [87]
Lignocerinic C24:0	0.40 ± 0.00	/	/
Saturated fatty acids	10.1 ± 0.28		
Monounsaturated fatty acids	75.8 ± 1.06		
Polyunsaturated fatty acids	13.7 ± 0.35		

**Table 3:** Phenolic compounds detected in the *Koelreuteria paniculata* seed cake ethanolic extract.

Compound	Content (μg/g)
Quercetin	5.11 ± 0.11
Quercitrin	14.28 ± 0.33
Gallic acid	749.32 ± 12.22
Ellagic acid	945.27 ± 16.98
Rutin	903.68 ± 19.21
Catechin	791.44 ± 13.24

**Table 4:** Limit of detection (LOD) and limit of quantification (LOQ) for analyzed compounds.

Compound	LOD (μg/mL)	LOQ (μg/mL)	Linear Range (μg/mL)	R^2^	Equation of the Calibration Curve
Gallic acid	6.25	18.50	15–800	0.9995	y = 7222.3x − 2.2
Ellagic acid	12.50	30.20	15–560	0.9998	y = 17369.0x + 81.4
Rutin	3.15	8.40	5–450	0.9995	y = 15485.0x − 159.5
Catechin	5.25	10.10	10–800	0.9999	y = 8655.4x − 15.5
Quercetin	3.45	5.65	5–450	0.9997	y = 26656.5x + 50.6
Quercitrin	5.25	15.10	5–450	0.9998	y = 17984.0x + 126.1

## Data Availability

All data are already present within the manuscript.
